# A Modified Experimental Hut Design for Studying Responses of Disease-Transmitting Mosquitoes to Indoor Interventions: The Ifakara Experimental Huts

**DOI:** 10.1371/journal.pone.0030967

**Published:** 2012-02-09

**Authors:** Fredros O. Okumu, Jason Moore, Edgar Mbeyela, Mark Sherlock, Robert Sangusangu, Godfrey Ligamba, Tanya Russell, Sarah J. Moore

**Affiliations:** 1 Biomedical and Environmental Sciences Thematic Group, Ifakara Health Institute, Ifakara, Republic of Tanzania; 2 Department of Disease Control, Faculty of Infectious and Tropical Diseases, London School of Hygiene and Tropical Medicine, London, United Kingdom; 3 College of Medicine, Swansea University, Swansea, United Kingdom; 4 Faculty of Medicine, Health and Molecular Sciences, James Cook University, Townsville, Queensland, Australia; 5 Faculty of Science, Health and Education, University of the Sunshine Coast, Maroochydore, Queensland, Australia; Louisiana State University, United States of America

## Abstract

Differences between individual human houses can confound results of studies aimed at evaluating indoor vector control interventions such as insecticide treated nets (ITNs) and indoor residual insecticide spraying (IRS). Specially designed and standardised experimental huts have historically provided a solution to this challenge, with an added advantage that they can be fitted with special interception traps to sample entering or exiting mosquitoes. However, many of these experimental hut designs have a number of limitations, for example: 1) inability to sample mosquitoes on all sides of huts, 2) increased likelihood of live mosquitoes flying out of the huts, leaving mainly dead ones, 3) difficulties of cleaning the huts when a new insecticide is to be tested, and 4) the generally small size of the experimental huts, which can misrepresent actual local house sizes or airflow dynamics in the local houses. Here, we describe a modified experimental hut design - *The Ifakara Experimental Huts*- and explain how these huts can be used to more realistically monitor behavioural and physiological responses of wild, free-flying disease-transmitting mosquitoes, including the African malaria vectors of the species complexes *Anopheles gambiae* and *Anopheles funestus*, to indoor vector control-technologies including ITNs and IRS. Important characteristics of the Ifakara experimental huts include: 1) interception traps fitted onto eave spaces and windows, 2) use of eave *baffles* (panels that direct mosquito movement) to control exit of live mosquitoes through the eave spaces, 3) use of replaceable wall panels and ceilings, which allow safe insecticide disposal and reuse of the huts to test different insecticides in successive periods, 4) the kit format of the huts allowing portability and 5) an improved suite of entomological procedures to maximise data quality.

## Introduction

To assess efficacies of house-hold mosquito control interventions, such as insecticide treated mosquito nets (ITNs) or indoor house spraying with residual insecticides (IRS), it is important to understand what happens to mosquitoes inside and around the dwellings in which these candidate interventions are located. Specifically, it is essential to know if the mosquitoes actually enter these huts, how long they spend inside the huts, whether they die inside the huts or after leaving the huts, and whether these mosquitoes successfully bite and take blood from persons inside these huts. The answers to all these questions represent efficacy of interventions against target mosquito species, and therefore influences the choices of vector control methods. Behavioural responses such as insecticide avoidance [1] and physiological events such as mosquito mortality, feeding or survival [1], [2], [3] are assessed and compared between houses with and houses without the intervention(s) being evaluated.

### Difficulties associated with using local human houses to evaluate efficacy of vector control interventions

Ideally, trials of household vector control tools should be conducted in actual human dwellings, where the relevant interventions are intended for use. However, there are many variations between individual houses, which can confound or even mask the real effects of candidate interventions being investigated. One common source of such variation is inconsistent number of house occupants and the associated differences in attractiveness of those occupants to host-seeking mosquitoes [4], [5], which means that even in the absence of any intervention, the number of mosquitoes entering any two different houses might be dramatically different. Another source of variation is type and texture of house construction materials. For example some huts may have mud walls instead of plastered walls, while others may have thatched roofs instead of iron sheet covered roofs, creating different micro-climates indoors and subsequently differences in mosquito densities within these houses [6], [7]. Substrates used for house construction or for wall linings can also affect persistence of vector control insecticides sprayed on these surfaces [8], [9].

Third is the number and sizes of available openings in different houses, particularly where houses are poorly constructed. It is well-established that house design is a significant factor affecting mosquito entry into human houses and that screening of house openings, such as doors, windows and eave spaces can reduce both mosquito densities, and malaria cases in these households [10], [11]. The fourth important factor is spatial location of houses relative to mosquito larval habitats, which also affects the relative numbers of mosquitoes entering houses. This phenomenon has been observed in numerous studies where mosquito densities in houses near breeding habitats were significantly higher than houses further away from the known larval breeding sites [12], [13], [14].

Other than these inter-house differences, there are also difficulties related to mosquito collection procedures inside local human houses, as well as cultural issues that can also determine acceptability of such entomological procedures. For instance, houses often have items such as cloths, pictures or other assortments of objects hanging on walls, which can be hiding places for mosquitoes and potentially limit effects of insecticidal applications [15], [16]. Any attempt to remove these items, prior to testing indoor interventions would not only cause inconveniences to household members, but retaining them would also limit chances of recovering mosquitoes especially those that are killed as a result of the indoor interventions. The artefacts would also provide mosquitoes many un-standardised surfaces where they might rest without being affected by a treatment, therefore biasing results. In some places it is culturally insensitive and considerably intrusive to collect mosquitoes in places such as people's bedrooms. Moreover, experience has shown that it can sometimes be mechanically impossible to fit standard mosquito traps onto windows or eaves of many of these houses without having to modify the openings or to minimise mosquito exit from cracks and holes on houses [17].

Early stage evaluations of most public health interventions require strict ethical guidelines to be followed [18]. Using experimental huts, occupied by volunteer adults who are fully informed of the risks and benefits associated with the study, therefore provides a way to avoid exposing the general public to any new interventions [19]. Also, in large scale evaluations such as randomised controlled trials, which are the gold-standard for public health decision making, it can be difficult to demonstrate a direct relation between health benefits (e.g. reduction in disease prevalence or incidences) and the vector control intervention introduced [20], [21]. This is because causal chains in many public health interventions are inherently complex, and are constantly modified by a myriad of factors in space and time [20]. Here also, experimental hut studies can be useful in demonstrating causal relationships and also characterizing various biological indicators of health benefit, albeit at small scale. For example, the huts can be used to directly observe and measure reductions in number of mosquitoes entering human occupied huts whenever an intervention is used inside that hut. Such an intermediate measurement, in this case reduced mosquito densities, can then be used to estimate likelihood of select interventions having epidemiological impacts at community level [22], [23]. Lastly, small-scale experimental hut studies are considered as a cost-effective intermediate stage between laboratory and community trials to rapidly and safely select only those interventions with proven entomological impact, for further large scale epidemiological testing.

All the challenges outlined above highlight the need for specially designed huts constructed to enable representative monitoring and evaluation of household interventions against wild populations of disease-transmitting mosquitoes [24]. Other than collecting mosquitoes from inside surfaces like walls, ceilings and floors, the huts may also be fitted with special interception traps so that mosquitoes can be monitored as they enter and also as they exit huts. The experimental huts are usually standardised in size and shape and are sometimes constructed such that they look as similar as possible to the local houses in the study village [25]. This requires that in the beginning, a survey of local huts is conducted to identify important attributes such as shape, area of sleeping quarters, common construction materials, as well as size and number of openings like windows, doors and eave spaces (ventilation gaps under the roofs of many houses in the tropics). Cultural preferences including whether residents fit roof ceilings or window curtains should also be assessed.

### A brief history of experimental huts and their applications in mosquito-related studies

In early 1940s, Haddow *et al*, conducted a series of experiments involving mosquito collections inside local houses in western Kenya [26]. They quickly noted several differences between individual local houses in the same study area, and as a result of these observations, they created specially designed huts with standardised sizes and surfaces for purposes of mosquito collections. Important features of these early experimental huts were as follows: 1) they were similar in size and shape to the local houses in the study area, 2) they all had exactly the same design so that it would be reasonable to compare mosquito catches between them, and 3) it was easy for persons to collect mosquitoes from all the inside surfaces of the huts, a requirement that was fulfilled by lining the inside walls with mud, covering the roof with a single-thickness hessian and using minimum furniture inside the huts. In addition, these experimental huts were windowless, had open eave spaces, tightly fitting doors and steeply pitched roofs to prevent rain draining inside. To attract mosquitoes, the Haddow *et al* huts were usually occupied by young local boys aged 10–12 years old [26].

After Haddow *et al* [26], several researchers began building on this work, leading to development of many early forms of experimental huts [24], including the mud-walled huts used by Muirhead-Thomson in Nigeria [27], [28], [29], [30] and its modifications, later used by Burnett in mid 1950s [31] and by Hocking *et al* [32] to test residual insecticides against malaria vectors. Many improved hut designs appeared in the 1960s during the first malaria eradication era [24], including those used by Rapley and colleagues, which were suspended on concrete bricks and surrounded by water channels to prevent predator ants from climbing in and feeding on captive mosquitoes [33]. Unlike the early Haddow *et al* huts [26] that had been used primarily to catch mosquitoes resting indoors, these new huts were now fitted with traps on windows to also sample exiting mosquitoes. These improved huts, and other later designs, also fitted with window traps, are now commonly known as the window-type experimental huts [24].

In mid 1960s, a new type of experimental huts, referred to as veranda-type hut, was pioneered by Dr. Alec Smith working at the Tanzania Pesticide Research Institute (TPRI) in northern Tanzania [34], [35]. Smith's huts were different from Rapley's huts in that other than having window traps on them, they were surrounded by screened verandas, in which mosquitoes were captured as they exited the huts. In experiments where a set of window traps were fitted to ordinary window-type huts and another set of window traps fitted onto veranda-type huts, leaving the verandas unscreened, it was concluded that presence of the verandas did not affect the total mosquito catches, nor the entry and egress patterns of mosquitoes [34].

Smith described the window-type experimental huts as being suitable for assessing mortality of malaria vectors, during evaluations of toxic insecticides but not evaluations of irritant insecticides, since mosquitoes irritated by insecticides would leave the huts earlier than normal and via any available opening including eave spaces. Such mosquitoes would thus go unaccounted for if window-type experimental huts were used [34]. He also noted that some non-malaria vector species such as *Mansonia uniformis* frequently exit huts through eaves as opposed to windows and are therefore best studied using veranda-type experimental huts rather than the window-type huts. Even then, the veranda-type hut itself did not completely solve this problem because of the way they are used; normally with two opposite verandas left open to let in mosquitoes, meaning that any mosquitoes exiting via eave spaces on these open sides still remain unaccounted for. This necessitated introduction of the inward and upward slanting barriers on top of the inside walls of veranda-type experimental huts: i.e. baffles that direct mosquito movement to allow mosquito entry but prevent exit. The barriers were originally truncated cones made of plastic mosquito gauze or wire mesh that slanted towards the apex of the roof at approximately 2 cm away from but parallel to the roofing [36]. These slanting baffles allowed mosquitoes to enter the huts through the eave spaces but restricted their exit through the same openings, even when highly irritant chemicals had been sprayed inside the huts [36].

At about the same time Hudson and Smith [37] developed another new hut with no verandas, but which instead was fitted with louvers angled at 53° so as to let in mosquitoes but minimise light that entered through the louvers. By attaching a window trap onto the east side of the hut, the mosquitoes were sampled while exiting towards the rising sun; and these catches multiplied by number of louvers so as to approximate total of mosquitoes entering the huts. This type of experimental hut was promoted mainly because it was simpler and cheaper to construct but also because it required simpler entomological collection methods [24], [37]. A recent modification of the louver hut is the west African design (also equivocally known as the “veranda trap hut”) developed at Institute Pierre Richet, in Côte d'Ivoire [38]. Mosquitoes enter these huts through louvers located on three sides and are trapped within the huts or in walled verandas fitted with a netted window located on the east side and closed with a drop cloth each morning.

Other more modern and innovative hut designs include the extraordinarily high Maya-style huts constructed by Grieco *et al*, to study behavioural responses of *An. vestitipennis* to insecticides in Belize [39]. These huts, had wooden plank walls and thatched roofs with apices rising as high as 4.5 m from the floors, thereby requiring raised walk-way, on which the person collecting mosquitoes would stand to inspect the high roof. These particular huts, like many earlier window-type experimental huts were also constructed in such a way that they could accommodate interception traps fitted on both windows and doors [39].

Most recently, portable wooden experimental huts have now been developed, which offer an added advantage of being easy to transport and to assemble onsite. These portable huts were originally used by Dr. Nicole Achee and colleagues in Belize, Central America, to recapture marked mosquitoes released at different distances [25]. With regard to construction materials and also dimensions of sleeping quarters, these huts were comparable to local village huts in the study area, in the central Cayo district of Belize. Portability was introduced by using a collapsible aluminium framework, allowing the collapse of the entire superstructure of the huts (including roof, gables and walls) by simply unbolting the metal bars in the framework. Furthermore, both the roof and the hut walls could be dismantled into 4 hinged units and 16 planks respectively, for loading onto transporter-trucks [25].

Here, we describe a new improved hut type, The Ifakara experimental hut, which encompasses several essential properties of the previous hut designs.

## Methods

### Description of the Ifakara experimental huts

#### Design, general characteristics and dimensions

**Figure 1:**
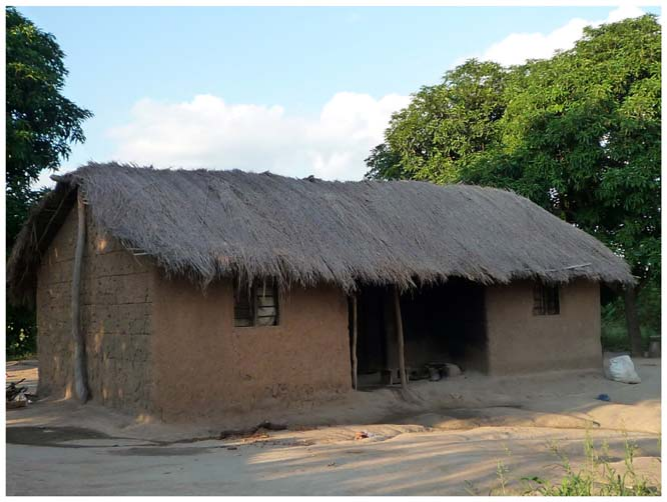
A typical local house used by communities in southern Tanzania. This example is from the area where the Ifakara experimental huts were first tested.

The Ifakara experimental huts are a new kind of hut, recently developed at the Ifakara Health Institute, Tanzania. The hut design encompasses proven merits of previous huts, but also aims to minimize some disadvantages associated with those previous designs. First constructed in 2007, these huts are already being used in Tanzania, Kenya, Zambia and Benin for various studies, including evaluation of LLINs and IRS (Okumu *et al* Unpublished), house screening against mosquitoes [40], mosquito repellents (Ogoma *et al* Unpublished), synthetic mosquito attractants [41] and mosquito killing fungal pathogens [42]. The original design of these huts was created to incorporate the portability principles earlier described by Achee *et al.*, [25]. However, with regard to shape, average dimensions and inside surface linings, the Ifakara experimental huts are similar to local village houses in rural communities in south eastern Tanzania, where these huts were originally used (Figure 1). It had been directly observed that local houses in Tanzania were mainly mud or brick walled, with thatched roofs [43]. However over the past three years, the proportion of roofs constructed from iron-sheet has increased to almost half [44]. Specific hut dimensions were collected using a housing survey in the study village.

**Figure 2:**
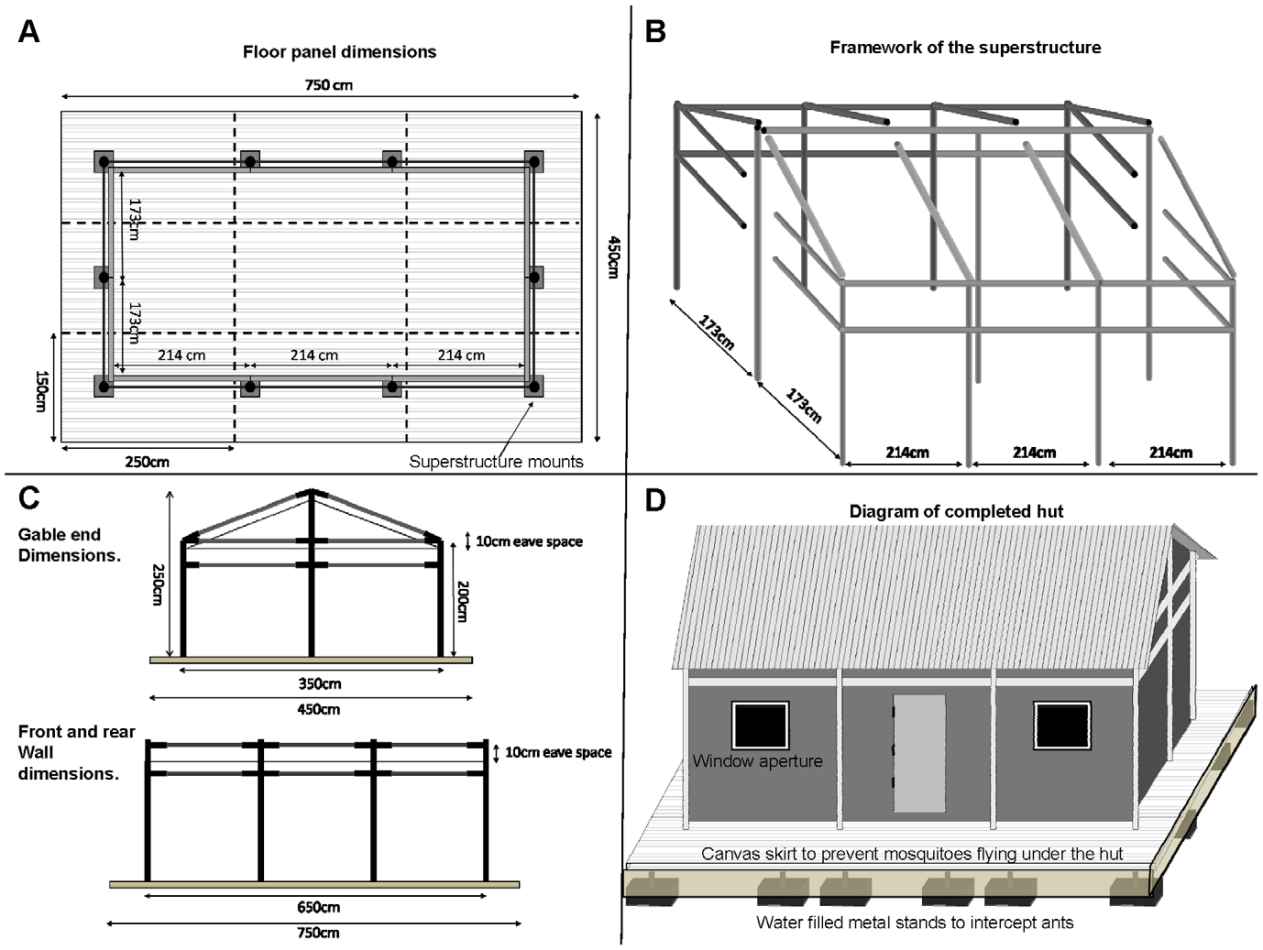
Diagrammatic representations of the Ifakara experimental hut designs. This figure shows the floor plan (panel A), the framework of the superstructure (panel B), side plans (panel C) and a complete view of the Ifakara experimental huts (panel D), showing important features.

**Figure 3:**
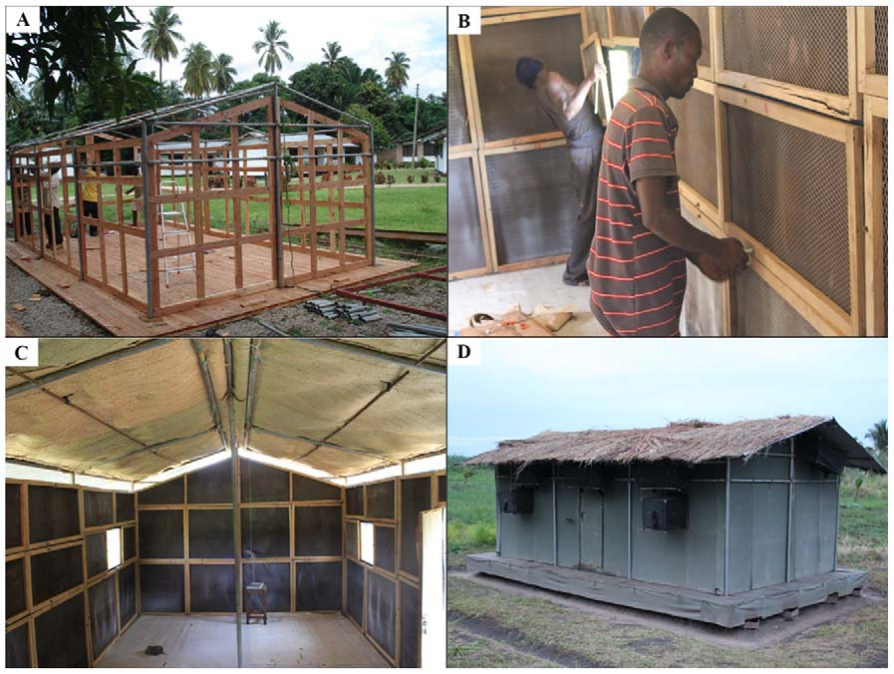
Pictorial representations of selected steps in the construction of the Ifakara experimental huts. Panel A shows the main framework of the Ifakara experimental huts under construction at the workshop. Panel B shows technicians fitting the wall panels, (which are made of chicken wire on wooden frames), onto the inside walls of the Ifakara experimental huts. Panel C shows the inside surfaces of the huts after fitting the chicken wire wall panels and also the palm woven (*mikeka*) ceiling on the underside of the roof, but before the inside walls are covered with mud, and Panel D shows a completed and functional Ifakara experimental hut, fitted with interception traps on windows and eave spaces. It should be noted that the overall shape and dimensions are set to match the typical local houses, shown in Figure 1. The hut is suspended on water-filled metal bowls to prevent predator ants, which would otherwise prey on the trapped mosquitoes.

Figures 2 and 3 show the framework and detailed dimensions, as well as important construction stages leading up to a finished Ifakara experimental hut. When completed, each hut covers a floor area 6.5 m in length by 3.5 m wide inside with a 50 cm walkway around the outside of the hut, and rises 2.0 m on the sides and 2.5 m to the apex of the roof. The huts have galvanized iron frames, with roofs made of corrugated iron sheets, which are overlaid with thatch to ensure that indoor temperatures do not vastly exceed the average temperatures inside local village houses (Table 1). The walls are constructed using canvas on the outside but are lined on the inside using removable wood panels that are coated with clay mud, which was the most common wall construction material used and found locally in the study area (Figures 1 and 3). The inside surfaces of the roofs are lined with woven grass mats, locally known as *mikeka*, and which also are common materials that local people use to make ceilings. Each hut has four windows (two on the front side and two on the back side) and one door (on the front side). For ease of transport and assembly on-site, the huts are designed and constructed in kit-format, with all individual pieces made in standardized sizes. Therefore despite the relatively large size, it takes 2 men, approximately 1–2 days to complete assembling one hut at a field site.

**Table 1:** Mean and standard deviations (SD) of daily temperatures and relative humidity (%) inside Ifakara experimental huts, as compared to local huts that have either grass thatched roofing or iron-sheet roofing. Data collected for 20 consecutive days in February 20011.

	Mean (SD) indoor temperature (°C)			Mean (SD) relative indoor humidity (%)		
	Local grass-thatched hut	Ifakara experimental hut	Local iron-roofed huts	Local grass-thatched hut	Ifakara experimental hut	Local iron-roofed huts
Day	26.5 (±1.3)	27.5 (±2.3)	29.2 (±2.4)	50.9 (±7.7)	87.9 (±9.3)	83.2 (±8.4)
Night	26.1 (±1.0)	25.1 (±1.7)	26.8 (±1.2)	51.1 (±8.7)	94.7 (±6.4)	89.4 (±4.4)

#### Features to prevent contamination when working with insecticides

To ensure that the main framework of the hut is never contaminated by any chemicals that may be used inside the huts or sprayed on the walls and ceilings (for instance when evaluating indoor house spraying with residual insecticides), continuous sheets of polyethylene (PE) are tightly fitted in the space between the outer framework of the huts and the mud panels and *mikeka* ceilings, which make up the insides hut surfaces. This PE sheeting, together with the mud panels and the *mikeka* ceiling, are not permanent components of the huts, and can be replaced whenever a new intervention or insecticide is to be tested in these experimental huts. The old materials can then be safely disposed of by incineration >1000°C using a T300 trench air burner (Air Burners LLC, FL, USA) available at the Ifakara Health Institute. Each Ifakara experimental hut has one door, four windows and an open eave space all round (Figures 2 and 3).

#### Features to prevent predation

To prevent scavenger ants from eating captive mosquitoes, the huts are suspended above ground using pedestals standing on water-filled metallic bowls (Figure 2D). The water in these bowls is regularly replenished and sprinkled with used-oil to also prevent mosquito breeding in them. Other than these measures, additional anti-ant precautions include regular cleaning of the huts, removal of shoes whenever one goes into the huts and clearing of all vegetation near and under the huts, which might otherwise be used by ants as a means to climb onto the huts (Figure 3D).

#### Features to prevent loss of mosquitoes

The huts are tightly finished and all individual pieces are well fitting, so that the only points for mosquito escape are windows and eave spaces, where interception mosquito traps are fitted. Any unwanted gaps around doors, eaves and windows are filled with hardened foam, to prevent mosquitoes that have entered the huts from escaping unaccounted for. As an additional precaution an oversized curtain can be hung on each the doors to prevent mosquito movement through the doors in case of accidental opening. The floors are covered with white, wipe-clean linoleum to ensure that any dead or knocked-down mosquitoes can be easily recovered. To minimize obstruction during mosquito collection, only the minimum essential furniture is kept inside the huts, i.e. two beds for sleeping volunteers and a ladder used during collections from the eave traps and ceilings. This practice, together with the lined inside surfaces and floors also minimize potential mosquito hiding places in the Ifakara experimental huts.

#### Traps and baffles used on the Ifakara experimental huts

**Figure 4:**
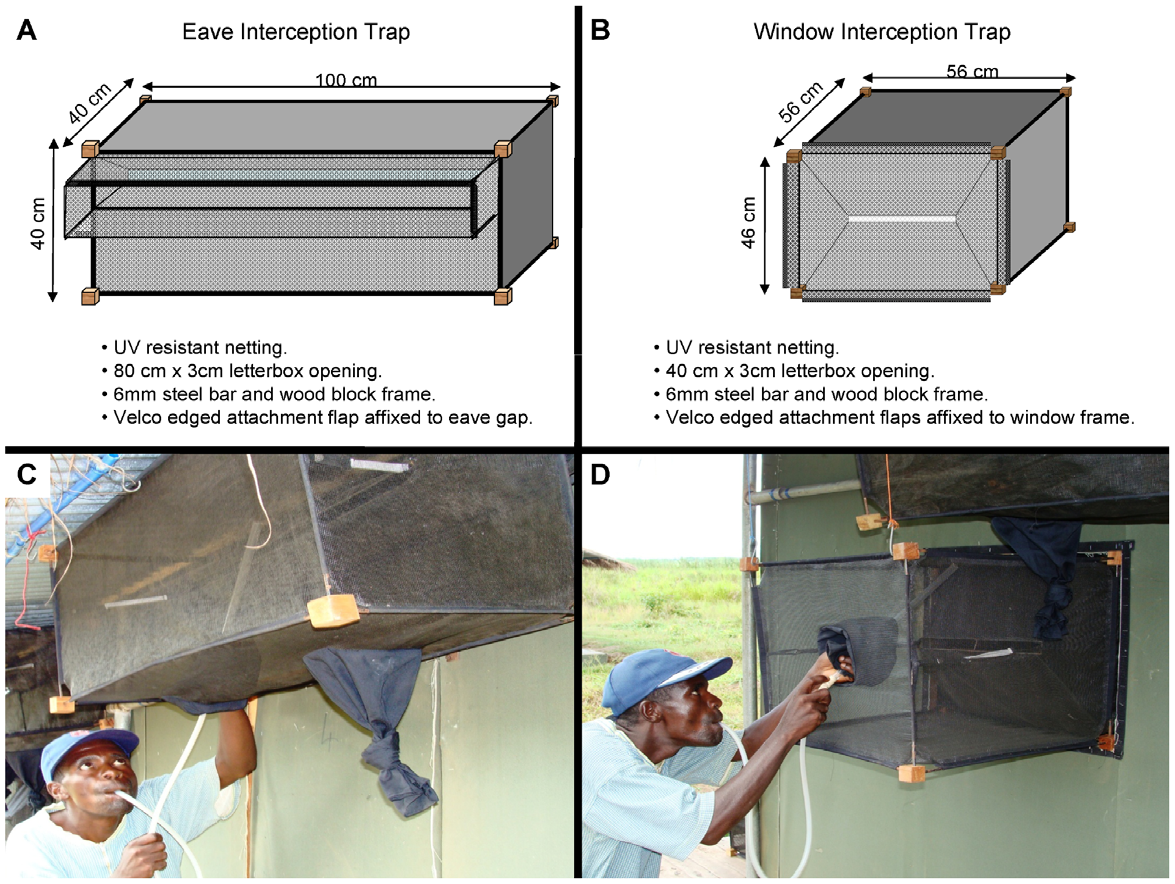
Diagrammatic illustration of eave trap and window trap. Panel A and B shows the dimensions and materials used to construct these traps, while panel C and D shows the eave and window traps fitted onto an Ifakara experimental hut during collection.

The huts are fitted with interception traps both on windows and eave spaces to catch mosquitoes. The designs and dimensions of these interception traps are illustrated in Figure 4. The versions presented here are the final result of a gradual trap development and improvement process, and should be considered as accessories of the Ifakara experimental huts, rather than as independent mosquito sampling tools. These traps can be fitted facing the inside of the hut to catch entering mosquitoes (in which case they are referred to as entry traps), or facing the outside so as to catch exiting mosquitoes (in which case they are referred to as exit traps). The entry and exit traps are specially designed to fit onto either windows (i.e. window traps) or on the eaves of the huts (i.e. eave traps), as depicted in Figures 3D and 4. In practice, the eave exit traps are therefore physically the same as eave entry traps, while the window exit traps are also physically the same as window entry traps. The traps are made of ultraviolet resistant shade netting (TenTex polypropylene net), mounted on a 5 mm wire frame, which is joined together using wooden blocks. The front end of each trap has a letterbox-shaped opening (measuring 80 cm by 3 cm on the eave traps and 40 cm by 3 cm on the window traps), to ensure that mosquitoes passing through the eave spaces or windows are let into the traps easily, but that these mosquitoes, once inside the traps cannot leave the traps as easily (Figure 4). To enable attaching onto the experimental huts, the netting with which the traps are made is extended to form attachment flaps specially fitted with Velcro-lined double seams. The frames of both window and eave spaces on all huts also have Velcro linings, so that the traps can be attached onto them. In this hut design, no traps are fitted onto the doorways, which instead are mostly kept shut except during passage of personnel. Moreover, we ensured that all the door shutters were tightly fitting and that there were no open spaces through which any mosquitoes could fly in or out. As such the only entry and exit points available for the mosquitoes were the eave spaces and windows.

**Figure 5:**
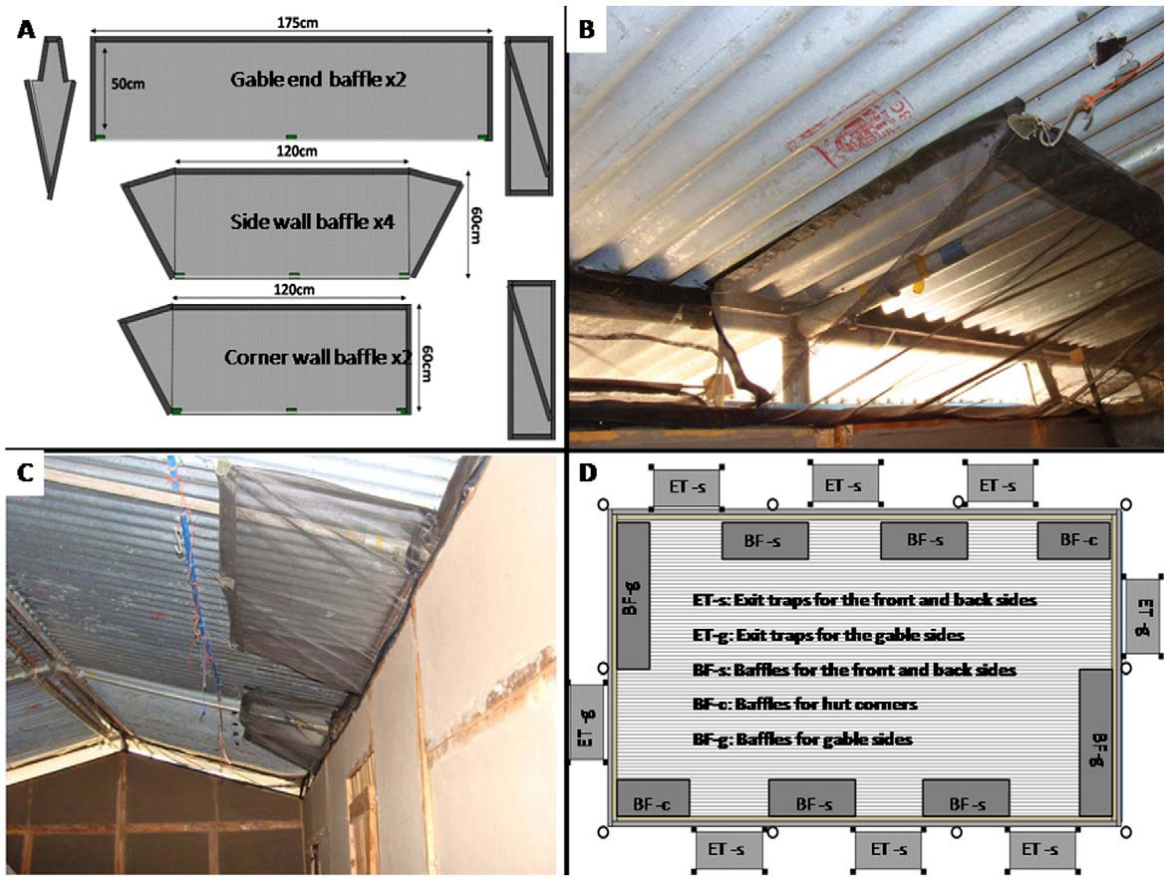
Netting baffles used in the Ifakara experimental huts. Panel A shows the design and dimensions of the different baffles used on front, back and gable sides, panel B and C are pictures showing two baffles fitted inside the huts and panel D shows the general layout of the baffles as interspaced with exit traps. Note that even though this diagram shows no *mikeka* ceiling under the roofs, the ceiling is an essential feature of all completed Ifakara experimental huts as shown in Figure 3.

Baffles on the other hand consist of upward-slanting and inward-facing netting barriers that are fitted on top of the walls of the experimental huts, so as to allow in mosquitoes, while at the same time preventing those mosquitoes that are already inside the huts from exiting via the same spaces (Figure 5). Netting was selected to encourage dispersal of human odour from the huts and therefore to maximise mosquito attraction to the huts [45]. The positions of the baffles on the eave space are interspaced between exit traps such that all mosquitoes that enter the huts can exit only via those spaces fitted with the exit traps (Figure 5C, D). The concept of interspacing baffles with exit traps all round the eaves also ensures that, similar to local human houses, there are adequate spaces through which mosquitoes can enter the experimental huts. It is expected that this practice removes directional bias, allows kairomones from human volunteers to be dispersed in a plume similar to that from a local house and maximises the spaces available for mosquito entry to maximise numbers in the huts. This is desirable in many field experiments involving free-flying wild mosquito populations, especially in areas where mosquito numbers are low, to improve the discriminatory power of the experiments. The baffles slant towards the apex of the huts and are held in parallel to the roofing using thin metal hooks (Figure 5B, C). There are two different sizes of these baffles, designed to fit onto either the gable side of the huts (175 cm by 50 cm baffles) or onto the long (front and back) sides of the huts (120 cm by 60 cm baffles). All baffles have Velcro-seamed ‘wing’ flaps, with which they are affixed to the roofs or walls of the huts, so that mosquitoes do not escape through the sides (Figure 5A, B).

In addition to mosquito collections using the interception traps, mosquitoes that enter the huts but fail to exit (e.g. fed mosquitoes resting indoors or those mosquitoes that are killed or knocked-down by insecticidal interventions) can be retrieved by direct indoor collections, from hut walls, ceilings or floors, using mouth aspirators. This procedure was implemented in the experiments conducted to test the experimental huts, as described later in this article.

#### Geographical positioning of the Ifakara experimental huts within the study area

To exemplify how best to spatially position these experimental huts during entomological studies, this section describes geographical sitting of nine Ifakara experimental huts, relative to the positions of local human houses in a rice growing village, in south eastern Tanzania, where we evaluated insecticide treated nets (ITNs) and indoor house spraying with residual insecticides (IRS) between 2009 and 2011 (Okumu *et al* Unpublished). The study site was in Lupiro Village (8.385°S and 36.670°E), Ulanga District. It lies 300 meters above sea level, and is approximately 26 km south of Ifakara town, where Ifakara Health Institute (IHI) is located. Although malaria transmission has been reducing steadily in this area [46], [47], [48], residents still experience perennially high transmission; latest estimates from neighbouring villages showing that unprotected individuals can still get as many as 81 infectious bites per year [46]. Malaria vectors in the area comprise primarily *An. gambiae* complex species, more than 95% of which are *An. arabiensis* [49], and a few *An. funestus* complex mosquitoes, 99% of which are *An. funestus* s.s. Giles (Okumu et al Unpublished).

**Figure 6:**
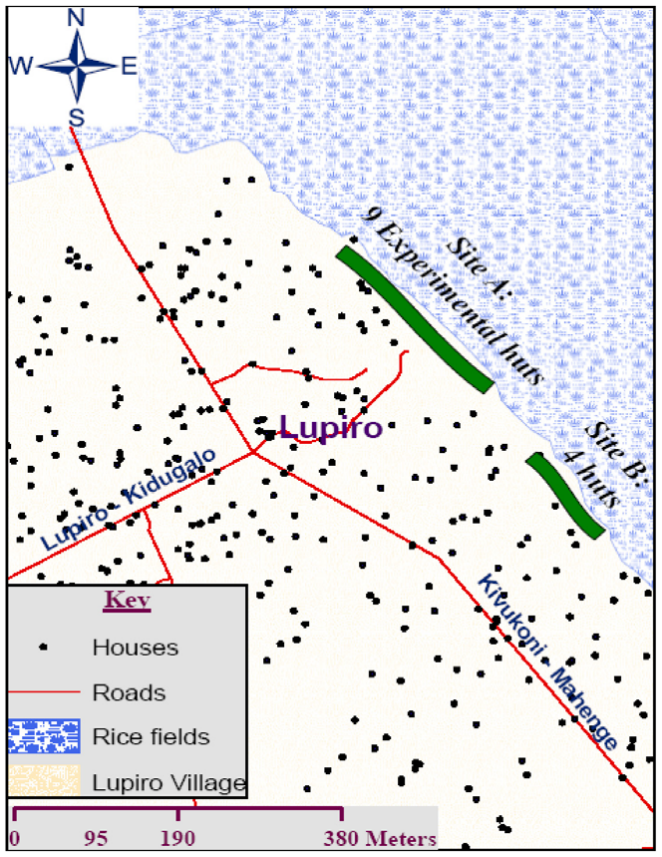
Geographical positioning of the Ifakara experimental huts. A map of the study area showing two sites at the edge of the village where Ifakara experimental huts are currently located. Site A has 9 huts while site B has 4.

The huts are located on a stretch of land at the edge of the village, such that that the huts are between the perennial irrigated rice fields (being the main larval mosquito habitat in the study area) and human settlements (Figure 6). For newly-emerged mosquitoes, this positioning enhances accessibility of these huts, relative to local houses. Considering natural dispersal patterns of mosquitoes over landscapes, and associated heterogeneities of their population densities [13], [14], it was envisaged that emergent host-seeking vectors from the irrigated rice fields are invariably more likely to first encounter these experimental huts, than the residential village houses, which are geographically farther from the breeding sites (Figure 6). Also, one other advantage of this positioning strategy is that even though our studies often involve large groups of volunteers and field assistants working in the huts at night, there is minimal disturbance to local villagers, since the huts are far from the main settlement area.

#### Climatic factors inside and outside the Ifakara experimental huts

To monitor the various climatic variables that may affect densities and/or behaviour of mosquitoes in the study site, an electronic weather station (LaCrosse Technology, USA) was positioned at the site, with an indoor sensor located inside one of the experimental huts. Using this wireless station, climatic variations were continuously recorded both indoors and outdoors on an hourly basis. These included indoor and outdoor temperatures and relative humidity but also wind speeds, wind direction, and rainfall. In addition, a set of portable data loggers (Tinytag Plus, TGP-4500) were introduced in two experimental huts and two local huts (one having a grass thatched roofing while the other having iron sheet roofing), so temperature and humidity changes could be directly compared between the hut types.

### Baseline studies using the Ifakara experimental huts: assessment of natural behaviour of mosquitoes in and around human occupied huts, and evaluation of a natural spatial repellent sprayed in the huts

Prior to testing any vector control technologies using the Ifakara experimental huts, studies were performed to understand how local mosquito vectors in the study area naturally behave in and around human occupied huts. It was also necessary to assess efficacies of both the baffles and the interception traps, as used on Ifakara experimental huts. The interception traps were evaluated in comparison to a standard entomological sampling method for indoor host-seeking mosquitoes, the Centres for Disease Control Light Traps (CDC-LT), set near a human volunteer sleeping under a bed net [50], [51]. This validation of efficacy of baffles and interception traps was performed using four experimental huts as described below. These initial studies also enabled us to trouble-shoot and to assess the utility of these huts for evaluating insecticidal applications such as LLINs and IRS.

#### Studies to determine: a) the times when local mosquito species normally enter human occupied huts, and b) the efficacy of entry traps relative to the standard, CDC-Light Traps

Four Ifakara experimental huts, each with 2 volunteers sleeping under non-insecticidal bed nets, were used. The four huts were paired, and in each pair one of the huts was fitted with entry traps on windows and on eave spaces, while the second hut had CDC-LT set up at a position between the two human volunteers sleeping under non-insecticidal bed nets, to catch mosquitoes entering the huts [51], [52]. The CDC-LT was fitted with timed bottle rotator (John Hock, FL, USA) to sample mosquitoes every hour. The volunteers stayed inside each hut between 7pm and 7am, during which time the traps were emptied each hour and all mosquitoes collected were aspirated into different paper cups, clearly labelled to show both the time of collection and type of traps used. Every night, the entry traps and the CDC-LT were rotated between individual huts in each pair of experimental huts. These cross-over tests were replicated 8 times over a period of 16 consecutive nights and each morning, all mosquitoes collected were sorted by taxa and their respective counts recorded.

#### Studies to determine: a) times when local mosquito species normally exit houses, b) efficacy of the exit traps and c) efficacy of the baffles fitted on open eave spaces of the Ifakara experimental huts

Four experimental huts, each with 2 volunteers sleeping under untreated bed nets, were used. On two of the huts, exit traps were fitted on 2 windows facing east with the other 2 windows open to allow mosquitoes to enter. Exit traps were also affixed to the eave spaces, interspaced with one-meter open spaces between them, as shown in Figure 5C, to allow mosquitoes to enter huts via the eaves. As a standard, CDC-LT was set inside the remaining 2 experimental huts [51], [52]. Since we also wanted to assess whether our baffles can indeed minimize possibility of mosquitoes exiting directly through the open eave spaces as opposed to flying into the exit traps themselves (Figure 5), two of the huts (one with exit traps and another with CDC-LT), were additionally fitted with the baffles.

The four treatments tested each night were therefore as follows: Treatment 1) one hut fitted with baffles and exit traps; Treatment 2) one hut fitted with baffles and CDC-LT; Treatment 3) one hut fitted with no baffles but with exit traps; Treatment 4) one hut fitted with no baffle but with CDC-LT. These treatments were rotated between huts on nightly basis, and were compared against each other in a 4×4 Latin square experimental design with each round replicated 4 times over a period of 16 consecutive nights. This experiment was repeated twice at different times. The volunteers stayed indoors between 7pm and 7am each night, and mosquitoes entering the huts were sampled hourly using the exit traps or the CDC-LT that was fitted with a timed CDC-bottle rotator (John Hock, FL, USA). The collected mosquitoes were aspirated into different paper cups, clearly labelled to show both the time of collection and type of traps used. Each morning, all the mosquitoes were sorted by taxa and their respective counts recorded.

#### Studies to: a) determine whether it is more efficacious to use both exit and entry traps on each experimental hut, relative to using just one trap type on the huts, and b) compare the number of mosquitoes entering the individual huts

We initially envisaged that by sampling exiting and entering mosquitoes in any given hut during the same night, we would significantly reduce potential biases possibly arising from daily variations of mosquito densities as well as wind direction. An experiment was therefore conducted in which individual experimental huts were fitted with either a combination of entry and exit traps, or with just entry traps alone or exit traps alone. Since this experiment involved mosquito collections in all the 9 experimental huts earmarked for our subsequent studies, it also enabled us to assess if there were any differences in numbers of mosquitoes entering the different individual huts in their designated locations.

Tests were conducted as follows: nine experimental huts were used, each with two volunteers sleeping under non-insecticidal bed nets. Each night, three of the nine experimental huts were fitted with a mixture of entry and exit traps (Treatment 1), another three were fitted with entry traps only (Treatment 2) and the remaining three fitted with just exit traps only (Treatment 3). Whenever the exit traps were used, and also whenever a mixture of entry and exit traps were used, baffles were fitted on the open eave spaces to prevent mosquitoes from exiting the huts via spaces other than those fitted with exit traps (Figure 5). In the three huts with mixtures of the entry and exit traps, the different trap types were interspaced so that any two opposite sides of the huts had equal number of entry traps or exit traps.

The trap arrangements were rotated weekly in such a way that at the end of the 3-week experiment, each hut had been fitted with each arrangement for one week (working for six nights a week). Due to logistical difficulties, the entry and exit traps were emptied three times a night at 11.pm, 3.00am and 7.00am, as opposed to hourly as in the previous experiments. To ensure that the total number of mosquitoes entering each hut was accounted for, further collections were conducted each morning from the inside hut surfaces using mouth aspirators, to retrieve any mosquitoes that had entered the huts during the night but failed to exit. The mosquitoes collected from each hut were aspirated into different paper cups, clearly labelled to show time of collection, trap from which the mosquitoes originated and trap arrangement used on the hut. Each morning, the mosquitoes were sorted by taxa and their respective counts recorded.

#### Studies to troubleshoot and optimize operations involving application of insecticides in the Ifakara experimental huts

Prior to introduction of any insecticidal applications in these huts, studies were conducted in which a behaviourally active test compound was applied on the mud panels of the experimental huts (Figure 3). A botanical mosquito repellent, para-methane 3,8 diol (PMD), which does not have long-term residual effects, was selected for this purpose [53], [54]. The low-residual property was particularly important so that the test compound would not confound effects of any other insecticidal applications used in the experimental huts at a later date.

This step enabled us to identify any potential limitations of the huts and vital adjustments necessary, meaning it was essentially a troubleshooting and optimization process, with a secondary objective of evaluating effects of PMD on behaviour of local mosquitoes. Specific activities that required trouble shooting included, spraying techniques, hourly mosquito collection, data management techniques, ways of addressing important volunteer needs, and other minor logistical challenges such as dealing with accidental scavenger-ant invasion in the experimental huts.

**Figure 7:**
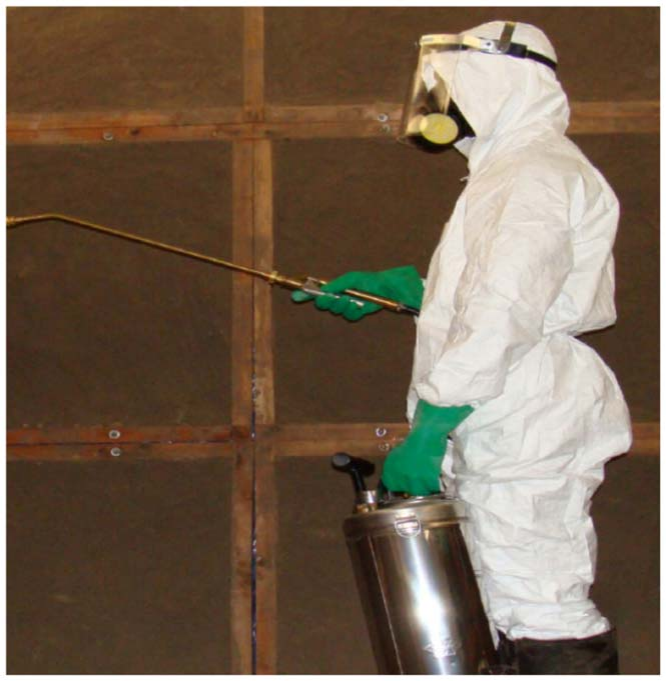
Spraying inside the experimental huts. Picture of a fully suited spray person applying PMD onto inside walls of the Ifakara experimental huts using standard Expert Hudson™ sprayers.

Four experimental huts each with 2 volunteers sleeping under untreated bed nets were used. Two of the selected huts were treated with PMD at a concentration of 1 gm^−2^ sprayed on the hut walls. PMD is not typically sprayed on walls so the concentration was based on laboratory data of relative repellency compared to DDT as a standard (Dr. John Grieco, personal communication). Once the target doses of PMD were calculated, the total amount of PMD required per hut was weighed and thoroughly diluted in the correct volume of water predetermined to cover the entire internal wall surfaces of the huts. The spraying was performed using standard Hudson Expert™ sprayers as illustrated in Figure 7. The other 2 huts were left as controls and were sprayed with only water. The four experimental huts were paired so that each pair had a PMD sprayed hut and a control hut to be directly compared against each other in two cross-over experiments as follows: Huts in the first pair were fitted with entry traps on windows and eaves to catch mosquitoes while entering huts. On the other hand, huts in the second pair were fitted with exit traps on windows and eaves to catch mosquitoes while leaving the huts. Baffles were added in the second pair of huts to limit unmonitored mosquito exit through the eave spaces. None of the treated huts was re-sprayed during the entire experiment period, which lasted 6 nights. Given the said purpose of this experiment, we did not conduct any assays to determine residual content of the PMD on the sprayed walls, hence the experimental period was limited to only six nights rather than several weeks as is common practice in experimental hut evaluations of public health insecticidal applications [19].

Each night, the sleeping volunteers rotated between the two huts in each treatment pair of huts to eliminate potential confounding effects resulting from any differential attractiveness of volunteers to mosquitoes [4], [5]. The exit and entry traps were emptied hourly from 7pm to 7am and the collected mosquitoes from each hut were aspirated into different paper cups, clearly labelled to show the time of collection, the trap from which the mosquitoes originated and whether the experimental hut had been sprayed with PMD or not. In addition, to ensure that the total number of mosquitoes entering each hut was accounted for, further collections were conducted each morning from the inside hut surfaces using mouth aspirators, to retrieve any mosquitoes that had entered the huts during the night but failed to exit.

#### Identification of mosquitoes

Each morning, all the mosquitoes were sorted by taxa and their respective counts recorded. The malaria vectors, *An. gambiae* complex and *An. funestus* complex mosquitoes, as well as other *Anopheles* mosquitoes were first distinguished morphologically from Culicine mosquitoes of other genera found in the study area i.e. *Culex* species and *Mansonia* species [55]. Molecular analysis by way of multiplex Polymerase Chain Reaction (PCR) [56], was then used to distinguish between *An. arabiensis* and *An. gambiae s.s*, the most predominant members of the *An. gambiae* complex found in the study area. Although, no PCR analysis was done on *An. funestus* complex mosquitoes collected during these early studies, the procedure was later incorporated in our subsequent tests, where all mosquitoes in this complex were shown to be *An. funestus* s.s [57].

#### Data analysis

Data analysis was performed using SPSS version 16 (SPSS Inc. Chicago, USA).

Data were analysed with Generalized Linear models with a negative binomial distribution and a log link to account for the over-dispersed nature of mosquito count data. Since most of the experimental huts data was clustered in individual huts, between which different treatments were rotated in a complete randomized block design, hut was included as a factor variable in all analyses. All models contained an intercept. Robust standard errors were used to account for any correlation between observations within huts.

When comparing mosquito catches related to any two categories (e.g. eaves trap vs. CDC-LT, or PMD sprayed hut vs. unsprayed hut), the regression intercepts were calculated and then exponentiated (as data were on a log scale) so as to enable the determination of efficiency of one treatment relative to an indicator variable reference, normally the control. Effects of the PMD spray was estimated following the WHO standard methodology [19], as a percentage reduction in number of mosquitoes caught in the PMD sprayed huts relative to the number of mosquitoes caught in the control huts.

#### Protection of participants and ethics statement

Participation in all our hut studies was entirely voluntary and the volunteers could leave at will at any stage during the experiment. After full explanation of purpose and requirements of the studies, written informed consent was sought from each volunteer prior to the start of all experiments. All participants received nightly wages as an incentive and to compensate for their time. Only males over 18 years were recruited as there are cultural implications of women working at night, and also ethical implications of recruiting women of childbearing age to a study where malaria infection could occur. Volunteers sleeping inside Ifakara experimental huts use intact bed nets so as to prevent mosquito bites. This is a minimum acceptable protection for research conducted in studies involving wild, potentially infectious mosquitoes, and was used in all cases as the universal experimental control when evaluating any candidate insecticidal applications. The volunteers were also provided with access to weekly diagnosis for malaria parasites using rapid diagnostic test kits and treatment with the first-line malaria drug (artemether-lumefantrine) in case they contracted malaria. Fortunately, none of the volunteers became ill during the period of these experiments. The study was approved by the Institutional Review Board of the Ifakara Health Institute (IHRDC/IRB/No. A019), the Tanzania National Institute of Medical Research (NIMR/HQ/R.8aNo1.W710) and the London School of Hygiene and Tropical Medicine (Ethics Clearance No. 5552).

## Results

### Climate measurements inside and outside Ifakara experimental huts and local houses

Indoor temperatures were similar between Ifakara experimental huts and the local grass thatched houses in the study village both during the day and also during the night. One way analysis of variance revealed a no significant difference in indoor night temperatures (F = 0.069, DF = 2, P = 0.998) between the huts, even though day time temperatures were significantly higher in local iron roofed huts than in both the Ifakara experimental huts and the local grass thatched huts (P<0.001). There was a significant a difference in relative humidity between local iron roofed huts and the experimental huts (F = 4.520, DF = 2, P<0.001), but not between the experimental huts and local grass thatched huts.

Tables 2, 3 provide a summary of climatic data at different times in 2010. As depicted by the standard deviations in Table 2, it is evident that for all of the important climatic factors, there were large variations during the daytime, but only minimal variations at night, when most of the mosquito collections were done. Also, we observed that even though it was warmer outdoors than indoors at daytime (average temperatures of 28°C versus 26°C), the huts were warmer than the outdoor environment at night (average temperatures of 23°C indoors versus 21°C outdoors). Similarly it was always more humid inside the huts than outside during the day (mean relative humidity of 66% versus 62% outdoors), but this was reversed during the nights, when it became more humid outdoors than indoors (mean relative humidity of 68% versus 84% outdoors). Finally, we also observed that winds were stronger and more variable during the day than at night, during which times the air was almost still (Table 3).

**Table 2:** Mean and standard deviations (SD) of daily temperatures and humidity inside and outside Ifakara experimental huts. Data collected between May and October 2010.

	Temperatures (°C)				Relative Humidity (%)			
	Indoors		Outdoors		Indoors		Outdoors	
	Mean	SD	Mean	SD	Mean	SD	Mean	SD
Day	25.85	3.96	28.13	5.33	66.49	9.99	62.28	18.42
Night	22.91	2.50	20.66	2.16	67.55	9.62	83.89	10.73

**Table 3:** Mean daily wind speeds and cumulative rainfall outside Ifakara experimental huts between May and October 2010.

	Wind speeds (Km/h)		Cumulative rainfall (mm)	
	Mean	SD	Mean	SD
Day	3.12	3.31	758.91	5.66
Night	1.25	2.44	758.81	5.69

### Molecular analysis of mosquitoes

PCR analysis of the *An. gambiae* s.l samples from the field studies showed that among the 1524 successful individual mosquito DNA amplifications, 96.7% were *An. arabiensis* (n = 1474) and 3.3% were *An. gambiae s.s* (n = 50). No molecular analysis was conducted for the other malaria vector, *An. funestus* complex mosquitoes, a few of which were also caught during these studies.

### Entry and exit behaviour of local malaria vectors in the study area

It was determined that the main malaria vector in the study area, *An. arabiensis* prefers to enter houses via eaves but to exit via windows, and that these mosquitoes exit houses mainly in the early morning hours between 3.00am and 7.00am. The number of mosquitoes entering huts at different times was generally equal throughout the night except for two small peaks, the first between 10pm and midnight and the second slightly more pronounced peak between 3am and 5am.

### Effects of baffles on exiting mosquito catches

Addition of the inward facing netting barriers (baffles) to the eave spaces of the experimental huts ensured that greater proportions of mosquitoes that entered the huts were retained and captured in the exit traps (Table 4). The trap catches were higher whenever baffles were used in the experimental huts relative to when no baffles were used. When data were aggregated by hut and day, the presence of baffles increased the number of *An. arabiensis* collected from a geometric mean (95% CI) of 64.68 (45.35–92.24) to 96.27 (69.79–132.81). This increase was statistically significant for *An. arabiensis*, relative rate (RR) 1.44 (1.17–1.77), z = 3.46, p = 0.001, and total mosquitoes collected RR (95% C.I.) 1.38 (1.10–1.73), z = 2.82, p = 0.005. When data for each trap type was analysed the use of baffles increased the likelihood of *An. arabiensis* being trapped in a window exit trap RR (95% C.I.) = 1.57 (1.03–2.37), z = 2.13, p = 0.033; and more than doubled the likelihood of *An. arabiensis* being trapped in an eave exit trap RR (95% C.I.) = 2.90 (1.89–4.48), z = 4.84, p<0.0001. When used with baffles, the number of mosquitoes recovered from window traps is not significantly different from CDC light traps indicating good sampling efficiency. The data (Table 4) also confirms that, even though *An. arabiensis* prefers to enter huts via eaves spaces rather than window spaces, these same mosquitoes tend to exit huts mainly via windows as opposed to eave spaces. The catches in light traps with baffles were also higher, indicating that the baffles did not inhibit mosquito hut entry.

**Table 4:** a Geometric Mean (GM) number and the 95% confidence intervals (CI) of mosquitoes caught in the Ifakara experimental huts. whenever baffles were used compared to when no baffles were used.

	*Anopheles arabiensis*							Total mosquitoes						
	Without baffles		With baffles		Relative Rate			Without baffles		With baffles		Relative Rate		
Trap type	GM	CI	GM	CI	RR	CI	P	GM	CI	GM	CI	RR	CI	P
CDC	74.5	45.8 121.2	96.7	69.9–133.7	1.3	1.1–1.6	0.009	152.7	98.3–237.2	181.8	133.8–247.0	1.2	0.9–1.6	*N.S.
Window exit traps	35.3	16.5–75.3	61.0	31.7–117.2	1.6	1.0–2.4	0.033	72.8	45.1–117.5	123.0	81.6–185.4	1.6	1.1–2.3	0.009
Eave exit traps	5.89	3.4–10.0	15.4	8.9–26.5	2.90	1.9–4.5	<0.001	8.4	5.0–14.1	24.1	16.4–35.4	2.7	1.9–3.8	<0.001

a The Relative Rate (RR) and 95% confidence intervals (CI) of the mosquito counts were calculated from Generalized Linear Models.

*N.S refers to, ‘not significantly different’.

### Effects of para methane 3, 8, diol (PMD) on the number of mosquitoes entering the experimental huts

Table 5 shows a summary of mosquito catches in huts sprayed with PMD and huts left as controls over the 6 experimental nights. In huts fitted with entry traps, there was a 49% reduction in median number of *An. arabiensis* mosquitoes caught in PMD sprayed huts compared to control huts. Median catches of *Culex* mosquitoes were reduced by 43% and *Mansonia* species by 20% (Table 5). When this data was subjected to generalized linear models, we observed no significant effects of PMD spraying on catches of any of these species even though the relative rates of mosquito catches were conspicuously lower than 1. The RR (95% CI) of *An. arabiensis* catches in PMD sprayed huts compared to control huts was 0.48 (0.21–1.08), z = 1.78, df = 1, P = 0.075. Relative Rate for *Culex* mosquitoes was 0.80 (0.34–1.89), z = 0.51, df = 1, P = 0.610) and that for *Mansonia* species was 0.53 (0.22–1.23), z = 1.44, df = 1, P = 0.151). We observed no significant effect of huts themselves on number of mosquitoes caught. Interestingly, we observed no reduction due to PMD treatment in any of the huts that were fitted with exit traps (Table 4). This was true for *An. arabiensis* (RR = 1.08 (0.49–2.42), z = 0.19, df = 1, P = 0.845), for *Culex* species (RR = 0.82 (0.34–1.89), z = 0.46, df = 1, P = 0.643) and for *Mansonia* species RR = 1.19 (0.52–2.75) z = 0.41, df = 1, P = 0.678). However the overall exit trap catches in PMD huts was higher than in control huts, suggesting that the presence of PMD was irritating and forcing excess mosquitoes out of the treated huts. This irritant effect accounted for 15.5% excess exit of *An. arabiensis* mosquitoes, even though this was not a statistically significant increase relative to the control.

**Table 5:** a Median number of mosquitoes of different species caught in Ifakara experimental huts that were either sprayed with PMD or left unsprayed.

		*Anopheles arabiensis*		*Culex* species		*Mansonia* species	
	Treatment	Median (IQR)	Sum	Median (IQR)	Sum	Median (IQR)	Sum
**Huts with entry traps**	Control Huts	22.5 (7.5–71.3)	470	3.5 (1.0–10.8)	90	5.0 (3.0–14.5)	100
	PMD sprayed Huts	11.5 (4.3–31.0)	224	2.0 (0.0–8.8)	72	4.0 (3.0–6.0)	53
	% Reduction	48.9%		42.9%		20.0%	
**Huts with exit traps**	Control Huts	152.0 (109.5–212.0)	1889	10.5 (6.8–16.5)	174	10.0 (4.3–17.5)	124
	PMD sprayed Huts	175.5 (129.5–218.5)	2046	10.0 (7.3–14.0)	143	11.0 (9.0–16.0)	148
	% Reduction	0.0%		5.0%		0.0%	

a Values in parenthesis represent interquartile ranges. Percentage reduction of mosquito catches due to PMD, was calculated based on the median mosquito catches.

### Comparison of the number of mosquitoes caught while entering or exiting experimental huts fitted with entry traps or exit traps alone versus experimental huts fitted with both entry traps and exit traps

Trap arrangement (i.e. whether the huts are fitted with entry traps only or with a mixture of entry and exit traps) affected the number of mosquitoes caught, even though in some cases, these differences were only marginally significant. The number of *An. arabiensis* caught exiting the huts (i.e. exit trap catches) was higher in huts fitted with only exit traps than in huts fitted with a mixture of exit and entry traps (RR = 1.24 (0.98–1.57), z = 1.78, df = 1, P = 0.076). Similarly, when mosquitoes were caught while entering huts (i.e. in entry traps), *An. arabiensis* catches were higher when the huts had only entry traps compared to when the huts had a mixture of entry and exit traps (RR = 1.65 (1.12–2.45), z = 2.50, df = 1, P = 0.012). We observed similar differences but with more pronounced statistical significance levels for *Culex* and *Mansonia* species mosquitoes. Specifically, in exit traps, the relative rate of *Culex* catches in huts fitted with only exit traps compared to huts fitted with both exit and entry traps was 1.50 (1.20–1.88), z = 3.57, P<0.0001 and in entry traps the RR was 1.84 (0.95–3.54), z = 1.81, P = 0.071. In the same order, the RR for *Mansonia* species in exit traps were 1.80 (1.16–2.80), z = 2.61, P = 0.009 and 1.45 (0.88–2.41), z = 1.67, P = 0.149 in entry traps.

Overall, the entry traps caught only about one eighth of all mosquitoes of all species that were collected in exit traps. In huts having a mixture of entry and exit traps, 90.4% of the *An. arabiensis* were caught in the exit traps, 8.4% in the entry traps and only 1.2% inside the huts, having failed to exit. On the other hand, in huts with only exit traps, 98.4% were caught in the exit traps and 1.6% inside the huts having failed to exit. Table 6 shows a summary of mosquito catches (median, interquartile ranges and sum of mosquitoes of different species collected when huts were fitted with either one type of trap or with a mixture of entry traps (50%) and exit traps (50%).

**Table 6:** a Median number of mosquitoes caught entering or exiting huts fitted with different trap arrangements namely: 1) entry traps only, 2) exit traps only or 3) a mixture of entry traps and exit traps.

		Experimental huts fitted with entry traps only		Experimental huts fitted with exit traps only		Experimental huts fitted with a mixture of entry traps (50%) and exit traps (50%)	
		Median (IQR)	Sum	Median (IQR)	Sum	Median (IQR)	Sum
**Exit trap catches**	*An. arabiensis*	-	-	211.0 (142.5–323.0)	12,714	176.5 (91.3–267.0)	10,263
	*Culex* species	-	-	8.0 (5.0–11.0)	464	5.0 (3.0–8.3)	309
	*Mansonia* species	-	-	10.5 (6.0–19.0)	950	7.5 (4.0–12.3)	528

		Median (IQR)	Sum	Median (IQR)	Sum	Median (IQR)	Sum
**Entry trap catches**	*An. arabiensis*	25.0 (17.0–34.3)	1,575	-	-	13.0 (6.0–26.5)	952
	*Culex* species	2.5 (1.0–4.3)	189	-	-	1.0 (0.0–2.3)	103
	*Mansonia* species	5.0 (3.0–9.0)	363	-	-	3.0 (1.0–6.3)	250

a The median values shown here were calculated per hut per night. Values in parenthesis represent interquartile ranges.

### Comparison of the number of mosquitoes entering different experimental huts

Summaries of catches for the different mosquito species in the 9 huts tested here are included in Table 7. Differences in mosquito catches between the huts was analysed using generalised linear models (GLM) based on totals of mosquitoes caught per night per hut, fitted in a negative binomial distribution model with a log link function. Using either the first hut (hut 1) or the last hut (hut 9) as reference, we observed that *An. arabiensis* catches in all the other huts were always significantly different from these huts (z = 6.00, df = 8, P<0.001). This was also true for *Mansonia* species (z = 6.07, df = 8, P<0.001), but not for the *Culex* species (z = 3.62, df = 8, P = 0.108) collected in the huts.

**Table 7:** a Median number of mosquitoes collected per night in individual experimental huts.

	*Anopheles arabiensis*		*Culex* species		*Mansonia* species	
	Median (IQR)	SUM	Median (IQR)	SUM	Median (IQR)	SUM
**Hut 1**	44.0 (23.3–75.5)^a^	922	4.0 (1.0–5.3)	75	7.0 (5.0–12.3)	166
**Hut 2**	228.5 (44.0–409.0)^b^	4488	3.5 (2.0–8.3)	99	18.0 (8.8–43.3)	528
**Hut 3**	168.5 (25.0–223.0)^b^	2514	4.5 (2.0–6.3)	87	6.0 (4.0–9.3)	126
**Hut 4**	76.0 (31.0–165.8)^b^	1810	5.0 (2.0–10.0)	109	8.5 (5.0–13.0)	218
**Hut 5**	152.5 (32.5–213.8)^b^	2613	10.0 (2.8–15.0)	183	28.0 (9.0–33.5)	482
**Hut 6**	133.5 (34.3–186.3)^b^	2227	7.5 (6.0–10.3)	184	12.0 (8.8–17.3)	248
**Hut 7**	144.0 (44.5–256.8)^b^	2971	5.5 (2.5–7.3)	93	7.0 (4.8–12.5)	156
**Hut 8**	195.5 (29.8–338.5)^b^	3602	5.0 (3.8–10.0)	126	10.0 (1.8–13.0)	158
**Hut 9**	313.0 (54.8–435.8)^b^	4805	8.5 (4.8–12.3)	149	7.5 (3.8–12.5)	145

a Values in parentheses represent interquartile ranges. For *An. arabiensis* letters that differ denote statistical significance.

In order to identify the actual individual huts contributing to these significant differences, we conducted a univariate GLM on log transformed *An. arabiensis* catches, with *post hoc* analysis using Tukey's Honestly Significant Difference test. Whereas this test confirmed an overall significant difference between mosquito catches in individual experimental huts (F = 2.859, df = 8, P = 0.005), two important findings emerged. First, hut 1 and hut 9 were the most different from the others. And second, significant differences were evident only when we directly compared hut 1 versus hut 2 (P = 0.013) or hut 1 versus hut 9 (P = 0.004), but not between any other pair of huts (P>0.05). When we eliminated catches from huts 1 and hut 9 and conducted a separate GLM analysis on the rest of the data, there were no significant differences between huts for *An. arabiensis* (z = 3.13, df = 6, P = 0.133) and *Culex* species (z = 3.02, df = 6, P = 0.165) but not *Mansonia* species (z = 5.64, df = 6, P<0.001).

## Discussion

The design of Ifakara experimental huts has been accomplished by combining advantageous design elements from several experimental huts previously used in mosquito studies [24]. Moreover, this design is an attempt to improve upon limitations identified in many of those previous huts. The final design of these new experimental huts has incorporated: 1) improvements on actual physical structure to make them more representative of local houses, 2) mosquito trapping methods that maximise mosquito entry and recovery as well as representative assessment of mosquito exposure to insecticides, 3) improved geographical positioning of the huts within the study area to maximise mosquito numbers while minimising disturbance to local residents; and 4) a suite of customised experimental practices employed when working with these experimental huts.

Some of the practical advantages of these huts are: 1) they are made in kit-format and can therefore be easily disassembled, transported between different sites and re-assembled onsite, 2) the possibility to replace the mud panels and the ceiling, whenever a new insecticidal application is to done so that all insecticides may be disposed of safely, 3) their similarity in style and size to local houses commonly used in the study area, which effectively improves their representativeness and 4) the fact that these huts, despite being fitted with traps all-round, still have adequate spaces for mosquitoes to enter. The huts can accommodate two human volunteers, who can both act as baits to lure in mosquitoes but also as mosquito collectors thus improving attraction to mosquitoes and maximising recovery of mosquitoes. This is clearly reflected in the high numbers of mosquitoes including the malaria vector *An. arabiensis* recovered from huts on a regular basis during our studies.

In our preliminary behavioural assays, for which results have been presented here, we observed clearly that *An. arabiensis* prefers to enter huts through eave spaces, but that these mosquitoes exit mainly through windows. We expected however, that if chemical-based interventions with irritant effects are used inside the huts, the mosquitoes may be forced to exit the huts via any available and nearest exits including the eaves [34], [58], thus disrupting the natural exit pattern. As Ifakara experimental huts with baffles collect similar numbers of mosquitoes in exit traps as CDC LT, these specific challenges have been overcome in the design. Results of these experiments evidently show that the baffles indeed boost exit trap catches, by retaining mosquitoes, which would otherwise exit unmonitored. It is also important to note from these results that presence of the baffles did not in anyway alter the entry pattern or the number of mosquitoes that entered the experimental huts.

Clearly, when evaluating household insecticide applications, these baffles become an even more important component of experimental huts, since they also guard against possible overestimation of percentage mortality due to candidate interventions. It is known that irritated mosquitoes tend to exit experimental huts through any opening including eave spaces [34], meaning that where there are no baffles, the sum of remaining mosquitoes, which is normally used as the denominator when calculating percentage mortality [19], will obviously be less than total number of mosquitoes that actually entered the huts. A good example of this can be found in early reports of work done by Dr. Alec Smith in northern Tanzania [35]. In one study investigating effects of an insecticide, dichlorvos, on mosquitoes visiting experimental huts, he observed that whenever mosquitoes leaving huts through the eave spaces were considered in his equations, the calculated mortality was always lower than whenever eave egress fraction was ignored [35]. Even with purely toxic and non-irritant insecticides, only the live mosquitoes would have a chance to escape, thus leaving mostly knocked down or dead ones inside the huts, a situation which can lead to an overestimation of proportions mosquitoes that die inside the huts, as a direct result of the insecticidal intervention being evaluated [34], [35], [36]. Therefore, we strongly suggest the use of baffles when evaluating insecticides in experimental huts.

In addition to the baffles, mosquito collection from all four sides of the huts on any given night, has some advantages over collection from only two opposite sides, which has been a common practice in previous studies involving veranda-type experimental huts [19], [34], [59], [60], [61]. This way, biases that may result from differences in directions of wind and light are minimised. Moreover, researchers also eliminate potential statistical problems associated with the previous practice of doubling the number of mosquitoes caught, so as to obtain the sum of mosquitoes that could have visited the huts if the collections were conducted on all sides of the huts [59], [60], [61]. Indeed, we have directly observed in our study area that this practice could be invalid, since the numbers of mosquitoes entering huts through any two opposite sides are never equal and in experiments where baffles were not used loss of mosquitoes is also not be equal on any two opposite sides, or exactly half of total entry.

Similarly, sampling mosquitoes on all sides, ensures that the open areas available for mosquitoes to enter the experimental huts is greater than seen among other hut designs, especially those previously used in west Africa, which allow mosquitoes to enter only via very small, 1 cm wide, window slits on three sides of each hut [62], [63], [64]. Again, we have demonstrated in our study sites in south-eastern Tanzania, that the malaria vector *An. arabiensis* prefers entering houses via eave spaces rather than through windows [40], but also that more mosquitoes enter huts if a greater area of the eave space is left unobstructed. This may suggest that the common west African experimental hut design such as the ones used in Benin [62] may not necessarily be as suitable for studying this East African vector population, as they have been for west African mosquito populations.

Another factor that has been addressed by the design described in this paper is prolonged mosquito retention within exit traps. It was observed during some early hut studies conducted in the 1960s that whenever mosquitoes were confined for long periods inside exit traps attached to insecticide treated experimental huts, there was excess mortality of mosquitoes in these traps, presumably due to concentrated fumes of the insecticides or accumulated insecticide dust deposits inside these traps [65], [66]. Despite these early observations, a common practice in current experimental hut studies is that mosquitoes remain held for long hours inside the exit traps or in verandas, and are removed only in the morning [19], [24], potentially increasing the probability of death as a result of this extended exposure to insecticide fumes.

One solution earlier proposed by Smith and Webley in 1963, was that insecticide-proof materials such as transparent polythene sheeting could be used to cover the side of window traps facing inside the experimental hut [66]. As described earlier, the traps used on the Ifakara experimental huts are all made entirely of netting, and instead the possibility of excessive mortality is minimised by regularly emptying the traps several times each night, so that the mosquitoes do not remain confined inside the traps and in close proximity to any insecticide fumes that could be emanating from the houses. This is usually done every 1–4 hours depending on research questions and associated logistical constraints. Once removed from the exit traps the mosquitoes are immediately transferred to a field insectary, 100 m away from the experimental huts, where they are maintained on 10% aqueous solution of glucose and monitored, usually for 24 hours. Other than being merely an attempt to minimize excessive mortality, this practice of multiple collections per night also more representatively matches what free-flying wild mosquitoes do around houses in real life; given that any mosquitoes found in the exit traps, are those that would otherwise have escaped completely from the huts. Moreover, such multiple collections now make it possible to identify and quantify irritant effects of insecticides which induce mosquitoes to exit huts earlier than usual [1], [58]. In fact, in previous experimental hut evaluations of insecticidal interventions in Africa, the closest estimates of irritancy were those based on overall differences between proportions of mosquito catches that were found in the exit traps in treatment versus control huts, and that in most cases, no attempts were actually made to assess whether insecticides induced earlier exit than normal [67]. This modification to allow multiple mosquito collections each night is therefore an essential improvement specifically in relation to huts previously used within Africa, which did not consider this aspect.

The third important practice conducted as part of the assay is blocking of some hut windows during the day. This is normally done in order to minimise potential effects of wind, i.e. the likelihood that any insecticides sprayed inside the experimental huts can be gradually eroded and blown around by wind, leading to rapid decay of the desired efficacies of candidate residual insecticides, while at the same time accumulating the eroded insecticide particles inside exit traps attached to the huts. Though the Ifakara experimental huts have 4 windows all of which are fitted with interception traps, 3 of the windows are usually covered during the day using tightly fitting pieces of canvas. These canvas covers are placed from the inside of the huts, effectively blocking the front part of the window traps during the day. They are however removed every evening so that all the 4 window traps can be used to collect mosquitoes during the night. Again, other than minimising effects of wind, our direct observations confirm that this particular practice correctly matches what normally happens in most local houses in southern Tanzania, where at least some of the windows are kept partially covered with curtains or wooden shutters during the day, or the windows remain fully closed.

Lastly, we initially observed that every evening just before our experiments began there were already a number of mosquitoes inside the huts. Since no volunteers stayed inside the huts during the day, and because most of these early mosquitoes were unfed, it is possible that either the mosquitoes entered the huts to rest [68] or they were lured by residual odours left behind by volunteers from the previous nights, and entered the huts anticipating blood meals [45], [68]. Experimental evaluations should therefore involve not only night-time collections, but also daytime collections where possible. Though such daytime collections are nowadays hardly conducted in experimental hut studies [19], early hut practitioners paid great attention to mosquitoes resting inside huts during the day [26]. In Ifakara experimental huts, collections targeting mosquitoes that may have entered huts during the day are done every evening between 1800 Hrs and 1900 Hrs, just before volunteer sleepers enter the huts to begin the night time catches. When testing interventions such as ITNs, which can be rotated daily or weekly between huts, the time when these nets are put into designated huts, i.e. whether this is done in the mornings or in the evenings, must be carefully considered so that these daytime effects are attributed to the right net type. Here also, inclusion of day-time catches more representatively captures the ‘round-the-clock’ interactions between mosquitoes and insecticidal interventions, when used inside local homes, than the current practice of monitoring only those mosquitoes visiting experimental huts at night [19].

Experiments conducted using a mosquito repellent PMD [53], [54], verified the suitability of the Ifakara experimental hut design in studies to assess effects of various insecticidal compounds on malaria mosquitoes. By corroborating the reduction in number of mosquitoes caught inside PMD spayed experimental huts relative to unsprayed huts and by being able to monitor all mosquitoes coming and leaving the huts, the tests provided a useful opportunity for identifying limitations in our procedures and also the necessary adjustments prior to subsequent studies using these huts. For example, we proved that emptying the traps every four hours is logistically possible on a routine basis, and as such this procedure was adopted for subsequent experiments.

Other than these observations, this particular experiment itself demonstrated the necessary training required for both the field technicians and the participating volunteers, on a wide range of entomological procedures involved in experimental hut evaluation of insecticidal interventions. We must also point out at this stage that even though these preliminary tests were carried out using just PMD (selected because it is a botanical with no long-term residual effects [53], [54]), it is logical to infer from the process and also from the results that indeed, these huts can be used to evaluate different insecticidal applications including LLINs and IRS, which may not have exactly the same mode of action as PMD. For example certain insecticides commonly used in ITNs e.g. permethrin [69], [70], [71] and also insecticides used for IRS e.g. the pyrethroid, lambda cyhalothrin [62], [72], [73], [74] and the organochloride, DDT [59], [75], [76], [77], [78], are known to be not only toxic to mosquitoes, but also repellent and can be evaluated using these experimental huts. Given the specific reasons for using PMD in this study, we did not consider it essential to incorporate any assays to determine residual content of the compound on treated hut walls, and therefore we are unable to determine how its effects on mosquitoes would change over time.

One particularly crucial observation during this experiment was that while reduction in mosquito catches due to PMD could be readily detected in huts fitted with entry traps, this was not the case in huts fitted with exit traps, in which PMD related reduction was 0% for *An. gambiae s.l* and *Mansonia* mosquitoes, and only 5% for *Culex* mosquitoes. It certainly raises concern as to whether exit traps alone could be adequate to evaluate insecticides which also have these deterrent properties. However, because we also observed a minor increase in *An. arabiensis* catches inside exit traps fitted on PMD sprayed huts, relative to traps fitted on control huts, one would argue that exit traps are more suitable for measuring irritant effects of treatments upon mosquitoes that are already inside the huts, while entry traps are better when assessing how different treatments deter mosquitoes from entering the huts in the first place. The PMD repellence therefore can only be clearly observed if one considers entry trap catches, which however are evidently are only a small fraction compared to exit trap catches as the two methods do not have the same sampling efficacy. What is undoubtedly clear from this preliminary evaluation is that there is a significant difference in trapping efficiencies between exit traps and entry traps.

Whereas combination of entry and exit traps provides an opportunity to study both entry behaviour and exit behaviour of mosquitoes concurrently, thus avoiding nightly variations in mosquito catches, our tests showed that using all exit traps in each hut collects more mosquitoes than when a combination of entry and exit traps are used. Moreover, the number of mosquitoes entering the huts could be grossly underestimated if only the entry traps are used; since these traps capture only about 13% of all mosquitoes that actually enter the huts. These experiments also showed that most of the mosquitoes were caught in exit traps, even though there was no insecticidal application used in the huts. These findings suggest that in the absence of any intervention, exit traps are more efficient than entry traps, therefore rather than combining the trap types, it is better to use only exit traps, interspersed with spaces fitted with baffles. Given that variation (as depicted by interquartile ranges) were not different for the different trap arrangements, the assertion that it is better to use exit traps can be based only on improved catches, but not on the fact that such a practice would reduce data variability. Moreover, that assertion may not be interpreted to mean that exit traps are always better than entry traps in experimental hut studies. On the contrary, it should be noted that the type of interception trap to fit must be guided by whatever research questions are being addressed. Moreover, it should also be noted that that even though exit traps performed multiple times better than entry traps in this study, both trap types are actually physically the same, except that one type is fitted facing the inside of the huts (entry traps), while the other is fitted while facing the outside (exit traps).

Entry traps for example, may have lower trapping efficiencies than exit traps, but as depicted by our PMD test results, these traps are clearly better for assessing repellent effects of interventions, than exit traps. Exit traps on the other hand, if used together with baffles would be better for examining toxicity and irritant effects of interventions. Similarly, where the interest is to also determine the actual time when mosquitoes enter houses, then entry traps emptied frequently, say hourly would be more useful than exit traps, which do not account for mosquitoes dead or knocked-down within the huts. Nevertheless, where exit traps are used, it is necessary that additional collections are done indoors using mouth aspirators, to retrieve mosquitoes that fail to exit huts. All these are essential considerations when assessing house-hold level protective efficacies of interventions. Therefore, users of these experimental huts must ensure that the trap arrangement used suits the intended purposes

In experiments where mosquito catches were compared between the different huts, there was variation between huts in mosquito density. These differences may be related to either the positions of these huts [14] or to the differences in attractiveness of the human volunteer pairs who slept in the huts [4], [5]. One limitation of this experiment was that due to the need for logistical simplicity and statistical replication the human volunteers did not rotate between the huts. As such, hut plus the volunteers assigned to that hut were treated as a single source of bias and it is therefore difficult to identify the proportion of this effect that was actually caused by the positional differences between huts. Nevertheless, the advance knowledge of these differences was important in informing design of subsequent experiments, in which candidate insecticidal interventions and controls that could not be rotated (IRS) were now randomly assigned several huts to increase replication and where possible, treatments (LLINs) rotated between huts at different positions, while retaining the volunteers in their respective huts.

One of the primary goals of the previous hut developers was to create huts that resembled local human houses, and the Ifakara experimental huts are therefore not the first huts to attempt matching designs of local houses in study areas. Nevertheless, we present these huts as an improvement relative to the existing hut designs, which arguably, did not fully achieve the goal of matching local houses. For example, the East Africa veranda trap huts are very small and would not necessarily have similar airflow as local houses [34], [60]. Similarly, the West African huts such as those used in Benin [62], allow mosquitoes to enter huts via very small slits on the sides, thus restricting the natural entry pattern and adjusting the airflow in the huts. Also the, way mosquitoes are collected in many of these existing huts, usually by retaining them in close proximity to the huts until morning, may not necessarily represent the natural behaviours of mosquitoes, especially where users are protected with nets. For instance, our own observation of *An. arabiensis* in this study site, suggests that when these mosquitoes enter huts where volunteers are protected with nets, they do not necessarily spend a long time inside those huts, but that instead, they readily exit the huts, presumably to continue host seeking elsewhere. Retaining the mosquitoes till morning in a veranda trap, would therefore possibly lead to longer exposure to whatever interventions are in applied in the huts. In light of the above examples, we recognize that though the Ifakara experimental huts may not in themselves be the perfect match to local houses they constitute an improvement towards this goal, especially since the existing east and west African hut designs have not been modified for many decades.

Despite these improved characteristics of the Ifakara experimental huts, we cannot at this stage propose this design as a replacement of any existing hut designs. We recognise that perhaps the most important issue in that regard is the need to directly compare different hut designs currently being used in Africa and assess their relative efficacies for assessing effects of indoor interventions on mosquitoes. Nevertheless, one must also consider the value of data that such comparisons would produce, and how generalizable the conclusions of any one study location would be to different locations, given the diversity of local house designs in Africa, but also the differences in house-entry and feeding behaviours of mosquitoes in different places. Moreover, since experimental huts that are currently being used have different functional mechanisms and sizes, and because it may not be possible to fit them with exactly the same types of interception traps, another challenge to direct comparison of hut types would be how to decide on output variable to measure, and how exactly that variable should be measured.

Therefore, even though this manuscript is limited to the description and preliminary testing of the Ifakara experimental huts as an alternative option when evaluating indoor interventions against East African mosquito populations, we strongly recommend that prospective users should independently assess the utility of the huts in their respective localities before using them. In addition, the entomological procedures described here provide a framework that may also be modified to more accurately match intended research purposes and to better evaluate effects candidate interventions being tested.

### Conclusion

The Ifakara experimental huts provide an improved system that can be used to realistically study the natural behaviour of wild free-flying populations of disease-transmitting mosquitoes, including the increasingly dominant African malaria vector, *An. arabiensis*, and to evaluate efficacy of various indoor vector control technologies. Their efficacy is enhanced by the improved design relative to previous hut designs, specifically the fact that mosquito entry is maximised to improve the power of evaluations. The huts use both eave and window traps thus making the design suitable for studying a wide range of mosquito entry and exit behaviours and the nature of traps fitted onto the traps, the use of eave baffles to control mosquito exit improves data reliability. The huts are designed to be an assay with the use of replaceable wall panels and ceilings, and the kit format of the huts, but also by the specific entomological practices used to sample mosquitoes in these huts.
